# Comparison of Intravitreal Anti-VEGF Agents With Laser Photocoagulation for Retinopathy of Prematurity of 1,627 Eyes in China

**DOI:** 10.3389/fmed.2022.911095

**Published:** 2022-05-31

**Authors:** Dandan Linghu, Yong Cheng, Xuemei Zhu, Xun Deng, Hong Yin, Yanrong Jiang, Mingwei Zhao, Xiaoxin Li, Jianhong Liang

**Affiliations:** ^1^Department of Ophthalmology, Peking University People's Hospital, Eye Diseases and Optometry Institute, Beijing, China; ^2^Beijing Key Laboratory of Diagnosis and Therapy of Retinal and Choroid Diseases, Beijing, China; ^3^College of Optometry, Peking University Health Science Center, Beijing, China; ^4^Xiamen Eye Center of Xiamen University, Xiamen, China

**Keywords:** retinopathy of the prematurity, type 1 ROP, APROP, anti-VEGF (vascular endothelial growth factor), laser

## Abstract

**Purpose:**

To compare the efficacies and treatment outcomes of intravitreal anti-VEGF agents and laser therapy in retinopathy of prematurity (ROP).

**Methods:**

A retrospective, non-randomized, comparative study of patients diagnosed with type 1 ROP or aggressive posterior ROP (A-ROP) treated with intravitreal anti-VEGF agents or laser therapy as primary treatment at the People's Hospital of Peking University.

**Results:**

A total of 1,627 eyes of 862 patients were included. In **Group 1**, 399 eyes of 204 patients were diagnosed with A-ROP or zone I type 1 ROP. The initial regression of the anti-VEGF subgroup was better than that of the laser subgroup, and the reactivation rate and rate of progression to retinal detachment were lower than those of the laser subgroup. In **Group 2**, 1,228 eyes of 658 patients were diagnosed with zone II type 1 ROP. The reactivation rate of the laser subgroup was lower than that of the anti-VEGF subgroup. No significant differences were found in the initial regression and the probability of developing retinal detachment. Among the anti-VEGF agents, the reactivation rate in eyes treated with conbercept was much lower than that in eyes treated with ranibizumab. The spherical power and spherical equivalents of eyes treated with laser were significantly higher than those of eyes treated with anti-VEGF agents 1 year after initial treatment.

**Conclusions:**

In contrast to laser therapy, anti-VEGF agents as primary treatments have potential advantages for eyes with zone I type 1 ROP and A-ROP. For eyes with zone II type 1 ROP, laser photocoagulation and anti-VEGF agents therapy showed similar efficacy; however, the rate of reactivation with laser therapy was significantly lower than that with anti-VEGF agents. Among the anti-VEGF agents, the reactivation rate was much lower in eyes treated with conbercept than in eyes treated with ranibizumab. Compared to anti-VEGF agents, laser treated eyes had greater trend to myopia.

## Introduction

Retinopathy of prematurity (ROP) is one of the leading causes of childhood blindness in both developing and developed countries and is associated with premature birth and oxygen intake (1). Aggressive retinopathy of prematurity (A-ROP) is considered to be a subtype in premature infants (2–5), characterized by rapid progression instead of following the typical stages of the disease.

Treatment of ROP has undergone changes in the last 30 years. Initially, studies (6–11) such as the CRYO-ROP trial (11) confirmed the effectiveness of cryotherapy. Later, studies (12–17) such as the Early Treatment for Retinopathy of Prematurity (ETROP) randomized trial (17) showed the efficacy of diode laser therapy. To date, laser therapy is still the classic treatment of ROP. With the in-depth study of the pathogenesis of ROP and many reports of the drawbacks of laser treatment of ROP, anti-VEGF therapy has also entered the treatment stage. A number of studies, such as the Bevacizumab Eliminates the Angiogenic Threat of Retinopathy of Prematurity study (BEAT-ROP study), has confirmed the efficacy of anti-VEGF agents in ROP treatment [bevacizumab (IVB) (18–21); ranibizumab (IVR) (22–24); conbercept (IVC) (25–27)]. In the last decade, the use of anti-VEGF therapy has increased significantly. However, considering the advantages and disadvantages of laser and anti-VEGF agents, the optimal treatment options remain controversial.

Our hospital is one of the first units to carry out ROP screening and treatment in China. We have experienced the evolution of treatments from external cryotherapy to laser therapy and then to anti-VEGF agents, including bevacizumab (IVB), ranibizumab (IVR) and conbercept (IVC). Here, we summarize our own ROP treatment data between 2010 and 2018 of cases diagnosed with type 1 ROP or A-ROP, treated with anti-VEGF agents (including bevacizumab, ranibizumab, conbercept) or laser photocoagulation primarily at our eye center, and followed for at least 6 months. In this study, we compared the efficacies and treatment outcomes of anti-VEGF and laser coagulation, including the rate of initial regression, reactivation requiring retreatment, retinal detachment, refractive status.

## Methods

This was a retrospective study that was conducted in a tertiary hospital, namely, People's Hospital of Peking University, Beijing, China. The research was approved by the Clinic Institutional Review Board and complied with the Declaration of Helsinki. All patients enrolled in the retrospective study were diagnosed with type 1 ROP or A-ROP, were treated with anti-VEGF agents (bevacizumab, ranibizumab, conbercept) or laser photocoagulation primarily within 72 h of diagnosis (A-ROP within 24 h) between 2010 and 2018 at the Eye Center in People's Hospital of Peking University, and had at least 6 months of follow-up. ROP was diagnosed and classified according to the International Classification of ROP (3). Type 1 ROP was defined as zone I any stage with plus, zone I stage 3 without plus, or zone II stage 2 or stage 3 with plus according to the Early Treatment Retinopathy of Prematurity Study (17). A-ROP was defined as increased dilation and tortuosity of the posterior pole vessels in all four quadrants with a new vascular network between vascularized and non-vascularized retina in zone I and posterior zone II (3). After we communicated with the parents of ROP infants, the parents choosed treatment method. For patients who cannot withstand general anesthesia, they can only be treated with anti-VEGF injection.

For anti-VEGF injection, topical anesthesia or inhalation anesthesia was used. The eyelid was opened with an eyelid speculum, 10% povidone-iodine was instilled, anti-VEGF agents [bevacizumab (IVB) at a dose of 0.625 mg/0.025 ml; ranibizumab (IVR) at a dose of 0.25 mg/0.025 ml; and conbercept (IVC) at a dose of 0.25 mg/0.025 ml, which was half of the adult dose for treating age-related macular degeneration (AMD)] were injected with a sterile 0.5-inch needle 1 mm posterior to the limbus.

For laser photocoagulation, inhalation anesthesia or intubation anesthesia were used. After an eyelid speculum was placed into the eyelid, an indirect laser was used to photocoagulate the entire avascular retina.

All the patients who underwent anti-VEGF injection or laser photocoagulation were reexamined the next day to assess infection, returned to our hospital 1 or 2 weeks (one week after the injection or 2 weeks after the laser therapy) to assess the efficacy of treatment, and then returned to the clinic according to the gestational age and eye condition as evaluated by our ophthalmologists. The supplementary treatment in the follow-up process included reinjection, laser treatment, external compression, vitrectomy with or without lensectomy.

In some patients, cycloplegic refraction 6 months after primary treatment was obtained during an examination under anesthesia.

The main outcome measures in this study included initial regression, reactivation requiring retreatment, and retinal detachment. Initial regression was defined as follows: plus disease or a ridge regressed partially or completely after the first treatment. Reactivation requiring retreatment was defined as plus disease or ridge reappearance; retinal detachment was defined as ROP worsening into stage 4a or 4b or stage 5 requiring external compression or vitrectomy with or without lensectomy.

The following information about the patients in this study was collected and recorded: gender, gestational age at birth, birth weight, ROP zone, ROP stage, plus disease, presence or absence of A-ROP, age at primary treatment, follow-up period, presence or absence of reactivation, and additional treatment.

### Classification of Patients

We classified all the eyes into 2 groups according to the ROP zone and ROP type: group 1 consisted of A-ROP and zone I ROP which meets type 1 diagnostic criteria, and group 2 consisted of zone II stage 2 or stage 3 ROP with plus disease. Each group was classified into two subgroups: one subgroup was treated with anti-VEGF agents, and the other was treated with laser photocoagulation. We compared the rate of initial regression, reactivation requiring retreatment, and rate of retinal detachment requiring surgery between the two subgroups in each group. In addition, refractive data was compared between these two treatments.

### Statistical Analysis

The data were analyzed using SPSS (version 22; SPSS Science, Chicago, IL). The Mann-Whitney *U*-test and Student's *t*-test were used to compare the quantitative data. Qualitative data were analyzed with the Chi-square test. A generalized estimating equation (GEE) method using the SAS procedure GENMOD (version 9.4, SAS Institute, Cary, NC) that allows for intereye correlation was used for analysis of binary treatment outcomes for zone II type ROP. Values of *p* < 0.05 were considered statistically significant.

## Results

A total of 1,627 eyes of 862 patients were included. **Group 1** contained 399 eyes of 204 patients, including 137 eyes of 71 patients with laser therapy and 262 eyes of 133 patients with anti-VEGF therapy. **Group 2** contained 1,228 eyes of 658 patients, including 266 eyes of 149 patients with laser therapy and 962 eyes of 509 patients with anti-VEGF therapy.

1. Comparison between anti-VEGF agents and laser therapy for ROP, including rate of initial regression, reactivation requiring retreatment and retinal detachment.

For **group 1 (A-ROP and zone I ROP)**, no significant differences were observed between the two subgroups in terms of gestational age, birth weight, sex distribution, postmenstrual age at treatment and follow-up period (shown in Table 1).The comparison of treatment outcomes were shown in Table 2; the initial regression of anti-VEGF subgroup was significantly better than that of the laser subgroup (*P* < 0.001), and the reactivation rate and the probability of developing to retinal detachment were significantly lower than those of the laser subgroup (*P* < 0.001, *P* = 0.001, respectively).

**Table 1:** Demographics of the infants enrolled.

	**Zone I ROP and APROP**			**Zone II ROP**	
	**Anti-VEGF group**	**Laser group**	***P*-values**	**Anti-VEGF group**	**Laser group**	***P*-values**
Patients, *n*	133	71	/	509	149	/
Male, *n* (%)	53 (39.8%)	31 (43.7%)	0.655	277 (54.4%)	87 (58.4%)	0.391
GA, weeks	29.5 ± 2.1	29.2 ± 2.1	0.246	28.7 ± 2.0	30.2 ± 2.3	<0.001
	(range: 25–37)	(range: 25–36)		(range: 20–36)	(range: 28–32)	
Bw, g	1334.7 ± 374.9	1282.2 ± 258.9	0.200	1252.5 ± 368.4	1410.9 ± 374.6	<0.001
	(range: 800–3,000)	(range: 850–2,035)		(range: 500–3,100)	(range: 850–2,700)	
PMA, weeks	36.5 ± 2.7	36.2 ± 2.3	0.427	39.7 ± 3.9	38.6 ± 3.2	<0.001
	(range: 30–47)	(range: 32–46)		(range: 28–57)	(range: 33–48)	
Follow-up months	20.3 ± 12.0	13.2 ± 1.6	0.063	17.8 ± 11.3	21.24 ± 18.7	0.044
	(range: 6.6–85.7)	(range: 9.47–107)		(range: 6.0–86.6)	(range: 6.0–83.0)	

*GA, gestational age; BW, birth weight; PMA, postmenstrual age*.

**Table 2:** The comparison of efficacies and treatment outcomes.

	**Zone I ROP and APROP**			**Zone II ROP**		
	**Anti-VEGF group**	**Laser group**	***P*-values**	**Anti-VEGF group**	**Laser group**	***P*-values**
Eyes, *n*	262	137	/	962	266	/
Initial regression-no. of eyes (%) regression	223 (85%)	97 (71%)	<0.001	933 (97%)	263 (99%)	0.406
Reactivation-no. of eyes (%)	123 (47%)	90 (66%)	<0.001	202 (21%)	21 (8%)	0.009
Retinal detachment-no. of eyes (%)	26 (10%)	30 (22%)	0.001	8 (0.8%)	3 (1.1%)	0.136

For **group 2 (zone II ROP)**, significant differences were observed between the two subgroups in terms of gestational age, birth weight, postmenstrual age at treatment and follow-up period, but the overall distribution was similar. The sex distribution showed no difference (shown in Table 1). Therefore, a GEE was used for multivariate analysis of the difference in initial regression, reactivation rate, and proportion of retinal detachment between the two subgroups. The comparison of treatment outcomes of group 2 is shown in Table 2. There was no significant difference in the initial regression or probability of developing retinal detachment between the two subgroups (*P* = 0.406, *P* = 0.136, respectively). However, the reactivation rate of the laser subgroup was significantly lower than that of the anti-VEGF subgroup (*P* = 0.009).

2. Comparison between the two anti-VEGF agents (ranibizumab vs. conbercept) for ROP.

A total of 916 eyes treated with intravitreal ranibizumab (IVR), 283 eyes treated with intravitreal conbercept (IVC) and 25 eyes treated with intravitreal bevacizumab (IVB) were included in our study. Among them, no significant differences were observed between eyes with IVR and those with IVC in terms of gestational age, birth weight, sex distribution, postmenstrual age at treatment and follow-up period. The comparison of treatment outcomes of IVR and IVC are shown in Table 3. No statistically significant differences in the initial efficacy or the rate progression to retinal detachment were found. However, the reactivation rate of IVC was lower than that of IVR: for zone I ROP and A-ROP, the reactivation rate of IVC was 23%, much lower than the 49% for IVR (*P* = 0.006). For zone II ROP, the reactivation rate of IVC was 12%, much lower than the 23% for IVR (*P* < 0.001).

3. Comparison of refractive errors between ROP patients treated with anti-VEGF agents and laser photocoagulation.

**Table 3:** The comparison of efficacies and treatment outcomes of IVR and IVC.

	**Zone I ROP and APROP**			**Zone II ROP**		
	**IVR group**	**IVC group**	***P*-values**	**IVR group**	**IVC group**	***P*-values**
Eyes, *n*	220	35	/	696	248	/
Initial treatment efficacy-no. of eyes (%) regression	187 (85%)	33 (94%)	0.091	675 (97%)	240 (97%)	0.781
Reactivation of ROP- no. of eyes (%)	108 (49%)	8 (23%)	0.006	160 (23%)	30 (12%)	<0.001
Retinal detachment- no. of eyes (%)	22 (10%)	2 (6%)	0.392	5 (0.7%)	3 (1.2%)	0.485

Refractive data were collected from 212 eyes of 110 ROP patients with regressed ROP 6 months after receiving laser or anti-VEGF injection. There was no significant difference in baseline data such as GA, BW, PMA between the two groups. In total, 121 eyes of 61 ROP patients received anti-VEGF injection only, and among them, 2 eyes had zone I ROP, 4 eyes had A-ROP, and the others had zone II ROP; the mean spherical refractive error was 1.51 ± 1.26 D, the mean astigmatism 0.79 ± 1.33 D, and the mean spherical equivalent was 1.90 ± 1.42 D. Ninety-one eyes of 49 ROP patients received laser therapy only, and among them, 4 eyes had zone I ROP, and the others had zone II ROP; the mean spherical refractive error was 1.61 ± 1.77 D, the mean astigmatism 0.65 ± 1.54 D, and the mean spherical equivalent 1.8 ± 1.99 D, No significant difference were found between these two groups (*P* = 0.617, *P* = 0.480, *P* = 0.691, respectively). However, refractive data from eyes of regressed ROP patients after 1 year of anti-VEGF injection or laser therapy were significantly different. The mean spherical refractive error of eyes (71 eyes/36 patients, 2 eyes had zone I ROP, 2 eyes had A-ROP, and the others had zone II ROP) treated with anti-VEGF agents was 1.20 ± 1.31 D; the mean astigmatism was 0.19 ± 1.51 D, and the mean spherical equivalent was 1.8 ± 1.99 D. The mean spherical refractive error of eyes (53 eyes/32 patients: 2 eyes had zone I ROP, and the others had zone II ROP) with laser therapy was 0.34 ± 1.16 D, and the mean astigmatism was −0.37 ± 1.62. Statistical differences were found between these two groups. The spherical and spherical equivalents were significantly higher in eyes treated with laser than in eyes treated with anti-VEGF agents (*P* = 0.06, *P* < 0.001, respectively). No difference was found in the power of astigmatism (*P* = 0.201). The above results are shown in Table 4.

**Table 4:** Comparison of refractive errors between ROP patients treated with anti-VEGF and laser.

	**6-month follow-up**			**1-year follow-up**		
	**Anti-VEGF group**	**Laser group**	***P*-values**	**Anti-VEGF group**	**Laser group**	***P*-values**
Eyes, *n*	121	91	/	71	53	/
Mean spherical refractive error (D) (D) eyes (%) regression	1.51 ± 1.26	1.61 ± 1.77	0.617	1.20 ± 1.31	0.34 ± 1.16	0.060
Mean astigmatism (D)	0.79 ± 1.33	0.65 ± 1.54	0.480	0.19 ± 1.51	−0.37 ± 1.62	0.201
Mean spherical equivalent (D)	1.90 ± 1.42	1.8 ± 1.99	0.691	1.8 ± 1.99	0.19 ± 1.53	<0.001

## Discussion

To the best of our knowledge, this is the largest sample size for a comparative study on the efficacy of intravitreal anti-VEGF agents and laser photocoagulation for ROP. As one of the earliest centers to carry out ROP screening and treatment in China, we reported the use of intravitreal injection of anti-VEGF agents or laser photocoagulation as first-line therapy among a total of 1,627 eyes of 862 premature infants from 2010 to 2018 in our center.

For our initial treatment options, we had used laser therapy for ROP patients before 2010, then IVB was started from 2011, IVR from 2011 and IVC from 2015. Most of the cases treated with laser and IVB happened before 2013. Later, as the anti-VEGF agents being used more widely in ROP, laser has been used relatively less frequently. However, in our center, for the patients who came from distant provinces or had difficulties in keeping long-term follow-up, we tended to choose laser therapy. In all of our cases, we had communicated fully with the parents of ROP patients, and the vast majority had chosen anti-VEGF treatment for its convenience, safety and efficacy. For ROP patients who could not tolerant general anesthesia, only anti-VEGF treatment could be selected. Thus, we observed that in zone II ROP, gestational age, birth weight and postmenstrual age in anti-VEGF group were all smaller than those in laser group. Moreover, for ROP cases accompanied by fibrous tissue on the ridge, we tended to choose laser instead of anti-VEGF injection, because our long-term clinical observations showed that in ROP cases with fibrotic proliferation, the treatment of anti-VEGF injection was more likely to aggravate retinal pulling and increase the risk of retinal detachment than laser therapy. This might be attributed to the role of anti-VEGF drugs played in fibrotic proliferation.

For our additional treatment options, the above factors were still very important. Besides, for ROP cases that recurred after first injection, if the avascular area was large, which meant the lesion was located posterior, we tended to choose anti-VEGF drugs because laser treatment would cause a definite visual field defect for patients. However, if ROP cases reactived after two injections, although there was still a large avascular zone, we would choose laser therapy.

Our clinical study showed the efficacies of both anti-VEGF agents and laser therapy as primary monotherapy for type 1 ROP and A-ROP.

1. anti-VEGF agents had advantages in zone I type 1 ROP and A-ROP.

Initial regression was an important observation indicator. In our study, for zone I ROP and A-ROP, the initial regression rate of the anti-VEGF agent group was 86%, which was obviously higher than that (71%) of laser ablation (*P* < 0.001). The initial response reflected whether the disease could be controlled quickly. It was well-established that anti-VEGF agents had a fast onset, whereas laser therapy worked relatively slowly. Moreover, the low initial regression rate of laser therapy for zone I ROP and A-ROP might be attributed to the fact that it was a more difficult operation than intravitreal injection. Pupil rigidity in some eyes would make it harder to expose the avascular area, which was much larger than that in eyes with zone II ROP, thus increasing the difficulty for laser surgery. Inadequate laser treatment could not effectively prevent ROP progression, so the rate of initial regression was low. Similarly, the sooner ROP disease was controlled, the better would be the prognosis. Our research had also confirmed this. Because of the fast-progression of zone I type 1 ROP and A-ROP, if the initial treatment could not control ROP in time, the rate of progression to retinal detachment in the anti-VEGF agent group was 10%, significantly lower than that (22%) in the laser group (*P* = 0.001).

Reactivation after the first effective treatment of ROP was also an important observation indicator. In our study, comparing the reactivation of eyes treated with laser or anti-VEGF agents, we found that in zone I ROP and A-ROP, eyes treated with anti-VEGF agents (47%) were less likely to experience reactivation than those treated with laser ablation (66%, *P* < 0.001), which was in accordance with the BEAT-ROP study (21) (3.2 vs. 35% for zone I ROP).

Therefore, from the three points of initial regression, the probability of progressing to retinal detachment and rate of reactivation, anti-VEGF agents had advantages in zone I type ROP and A-ROP when compared with laser therapy.

2. Laser photocoagulation and anti-VEGF agents therapy showed similar efficacy for eyes with zone II ROP, however, the rate of reactivation with laser therapy was significantly lower than that with anti-VEGF agents.

For zone II ROP, anti-VEGF agent group and laser ablation group both showed a high initial regression rate (97, 99%, respectively), which were much higher than those for zone I group and A-ROP (85, 71%, respectively), and no significant difference was found between the two groups (*P* = 0.406). This might be attributed to the relatively simpleness of laser operations for Zone II type 1 ROP. The sooner ROP disease was controlled, the better would be the prognosis. Because of the high initial regression of ROP cases in two groups, the rate of progression to retinal detachment were both low (anti-VEGF agent group 0.8%, laser group 1.1%) and no significant difference was found between them (*P* = 0.136).

However, the reactivation rate of eyes treated with anti-VEGF agents was 21%, which was much higher than those eyes treated with laser therapy (8%) (*P* = 0.009), which was opposite to the BEAT-ROP study (21) (anti-VEGF agents 5.1% vs. laser 11.2% for posterior zone II ROP). Unlike the BEAT-ROP study, reactivation requiring retreatment in our study was defined as plus disease or ridge reappearance, not neovascularization. Also, the composition ratio of ROP types was not the same. The BEAT-ROP study eliminated anterior zone II ROP, but we included all the zone II ROP, which met the type 1 ROP diagnostic criteria. As described above, the laser treatment for ROP which located posterior was relatively difficult, so the reactivation rate of ROP treated with laser in BEAT-ROP study was higher than that in our group.

Moreover, the ROP population was different. All individuals in the present study population were Asians, but those of the BEAT study were various ethnicities.

We believed that laser treatment in zone II ROP had a relatively high success rate, and it was easier to perform sufficient ablation. Once the peripheral retina was sufficiently photocoagulated, the damaged retina would no longer cause elevated VEGF concentrations in the eye. However, anti-VEGF agents had a metabolic cycle in the eye. During the continuous growth of the vasculature of the peripheral retina, the fluctuation of VEGF could still cause ROP reactivation, and therefore, the reactivation rate of the laser group was lower than that of the anti-VEGF agent group.

3. Reactivation rate of IVC group was lower than that of IVR.

A total of 916 eyes with IVR, 283 eyes with IVC and 25 eyes with IVB were included in our study. Among them, no statistically significant differences in the initial regression or rate of progression to retinal detachment were found between eyes treated with conbercept and those treated with ranibizumab. However, the reactivation rate of eyes treated with conbercept was much lower than those treated with ranibizumab. This might be attributed to the differences in structure of the two drugs. Ranibizumab is a well-established recombinant, humanized monoclonal G1 kappa isotype antibody Fab fragment that is structurally derived from the light chains of bevacizumab and is designed to bind all isoforms of VEGF-A. Conbercept is a recombinant soluble fusion protein composed of the second Ig domain of VEGFR1 and the third and fourth Ig domains of VEGFR2 with the Fc portion of human IgG (28). The receptor portion has a high affinity for all VEGF-A isoforms, PIGF 1 and 2, and VEGF-B (29). Most likely, because of more targets of conbercept than of ranibizumab, the effect of conbercept may be faster and more powerful. Thus, the reactivation rate in conbercept-treated eyes may be lower than eyes treated with ranibizumab.

4. The refractive results after 1 year of primary treatment showed that laser treatment had a greater effect on the change in refractive power than anti-VEGF injection had, and the refractive power was 1 D lower with anti-VEGF treatment than with laser treatment. Therefore, lasers were more likely to cause myopia and advances in pre-existing myopia, which were comparable with the effects observed in other studies. There was no difference in refractive data obtained over 6 months, which might be because the eyeball is still developing as the patient ages and the effect of laser on refractive power may gradually emerge.

In summary, our study demonstrated that for initial treatment in eyes with zone I ROP and A-ROP, anti-VEGF agents seemed to have potential advantages in contrast to conventional laser therapy. For eyes with zone II ROP, anti-VEGF agents and laser photocoagulation showed similar efficacy, however, the reactivation rate of eyes treated with laser therapy was significantly lower than those treated with anti-VEGF agents. Among the anti-VEGF agents, the reactivation rate of eyes treated with conbercept was much lower than those treated with ranibizumab. Lasers were more likely to cause advances in myopia.

## Data Availability Statement

The original contributions presented in the study are included in the article/supplementary material, further inquiries can be directed to the corresponding author.

## Ethics Statement

The studies involving human participants were reviewed and approved by Beijing University People's Hospital Medical Ethics Committee Peking University People's Hospital. Written informed consent to participate in this study was provided by the participants' legal guardian/next of kin.

## Author Contributions

JL: conceptualization, supervision, and writing—review and editing. JL and DL: methodology. JL, HY, MZ, YJ, and XL: cases provider. DL, YC, XZ, and XD: data collection and data curation. DL and YC: data formal analysis. DL: writing—original draft. All authors contributed to the article and approved the submitted version.

## Funding

This work was supported by the Beijing Science and technology project (Grant No. Z201100005520078), Beijing Bethune Charitable Foundation (Grant No. 2018-A-08), Beijing Science and Technology Planning Project (Z191100007619041), and National key R&D Program of China (No. 2020YFC2008200). The funders had no role in the study design, data collection and analysis, decision to publish or preparation of the manuscript.

## Conflict of Interest

The authors declare that the research was conducted in the absence of any commercial or financial relationships that could be construed as a potential conflict of interest.

## Publisher's Note

All claims expressed in this article are solely those of the authors and do not necessarily represent those of their affiliated organizations, or those of the publisher, the editors and the reviewers. Any product that may be evaluated in this article, or claim that may be made by its manufacturer, is not guaranteed or endorsed by the publisher.
